# Ellagitannins and Flavan-3-ols from Raspberry Pomace Modulate Caecal Fermentation Processes and Plasma Lipid Parameters in Rats

**DOI:** 10.3390/molecules201219878

**Published:** 2015-12-21

**Authors:** Bartosz Fotschki, Jerzy Juśkiewicz, Michał Sójka, Adam Jurgoński, Zenon Zduńczyk

**Affiliations:** 1 Division of Food Science, Institute of Animal Reproduction and Food Research, Polish Academy of Sciences, Olsztyn 10-748, Poland; j.juskiewicz@pan.olsztyn.pl (J.J.); a.jurgonski@pan.olsztyn.pl (A.J.); z.zdunczyk@pan.olsztyn.pl (Z.Z.); 2 Institute of Food Technology and Analysis, Technical University of Łódź, Łódź 90-924, Poland; michal.sojka@p.lodz.pl

**Keywords:** ellagitannins, proanthocyanidins, flavan-3-ols, microbial activity, HDL cholesterol, short-chain fatty acids, Wistar rats

## Abstract

Raspberry pomace is a source of polyphenols, which nutritional and health promoting properties are not sufficiently known. The aim of this 8-weeks study was to scrutinize if raspberry extracts (REs) with different ellagitannins to flavan-3-ols ratios might favorably affect the caecal fermentation processes and blood lipid profile in rats. Forty male Wistar rats were fed with a standard diet or its modification with two types of REs (E1 and E2) characterized by different ratios of ellagitannins to flavan-3-ols (7.7 and 3.1 for E1 and E2, respectively) and added to a diet at two dosages of polyphenolic compounds (0.15 and 0.30% of a diet; L and H treatments, respectively). Irrespective of polyphenols dietary level, both REs reduced the activity of bacterial β-glucuronidase, increased production of butyric acid in the caecum and reduced triacylglycerols in blood plasma. The E1 treatment at both dosages caused more effective reduction in the concentration of ammonia and elevated acetate level in the caecal digesta than E2. On the other hand, only the E2 treatment lowered value of the atherogenic index when compared with control group. When comparing dosages of REs, a higher one was more potent to reduce the activity of bacterial β-glucosidase, β-, α-galactosidase and lowered value of the HDL profile in plasma. To conclude, REs may favorably modulate the activity of the caecal microbiota and blood lipid profile in rats; however, the intensity of these effects may be related to the dosages of dietary polyphenols and to their profile, e.g., ellagitannins to flavan-3-ols ratio.

## 1. Introduction

Raspberries are one of the most widely consumed fruit in various forms, both fresh and processed. These fruits are known as a rich source of dietary antioxidants largely due to their high level of phenolic compounds, which are primarily comprised of cyanidins, anthocyanins, ellagitannins, conjugates of ellagic acid and quercetin [1,2]. In addition to their strong antioxidant properties, raspberries have also shown other beneficial bioactivities including anti-inflammation and anti-microbial activity against pathogenic intestinal bacteria [3]. To date, most raspberry-related studies have been focused on the oxidative stress and anti-cancer activity of raspberry juice and polyphenol extracts producing from whole fruits [4,5,6]. However, another valuable source of biologically active compounds seems to be raspberry pomace. During processing of the fruits, particularly in the production of concentrated juice, a significant part of compounds, mainly fiber and polyphenols, remain in the pomace, which nutritional and health promoting properties are not sufficiently known. The most frequently applied ways of recycling most of pomaces from fruits are for animal feed and composting or by disposal in dumps. Therefore, the need to study the exploitation, e.g., of phenolic by-products from raspberry processing, for the manufacture of functional additives to food is substantiated [6].

The main polyphenols presented in the raspberry pomace are ellagitannins, proanthocyanidins and anthocyanins [6]. These pomace phenolic fractions may play an important role in the inhibition of starch digesting enzymes as well as activity of the microbiota living in the lower gut. Moreover, supplementation with polyphenols may favorably reduce the plasma concentrations of total cholesterol, LDL-cholesterol, triacylglycerols (TG) and increase HDL-cholesterol concentrations plasma [7]. Some studies have shown that anthocyanins and ellagitannins could reach the lower part of the intestinal tract and there change microbial metabolism, and thus modulate fermentation in the gastrointestinal tract [8,9,10,11]. Also proanthocyanidins, belonging to the flavan-3-ols, have been shown to inhibit the activities of digestive enzymes and may have important local functions in the gut [7,12]. Our previous studies on rats have shown that polyphenol-rich extracts modulated the activity of gastrointestinal endogenous enzymes as well as production of short-chain fatty acids (SCFAs) which are a main end-products of the caecal fermentation [11,13]. A judicious level of SCFAs e.g., butyric acid, is profitable for mucosal cells and acetate seems to promote lipogenesis in the liver, whereas propionate has been reported to inhibit this process [11,14]. An undesired effect is posed to the whole organism by excessively high activity of microbial β-glucuronidase. This enzyme is characteristic for the harmful bacteria species and it has deconjugative properties which support the transformation of xenobiotics into more toxic substances [15,16]. The direction and rate of large gut fermentation processes might be modulate by dose and composition of dietary polyphenols [11,17].

The physiological status of the lower gut is known to be of paramount importance for the host organism. Therefore, the aim of this study was to scrutinize if raspberry extracts from pomace with different ellagitannins to flavan-3-ols ratios might favorably affect the caecal fermentation processes as well as blood lipid profile in rats.

## 2. Results

After eight weeks feeding with diets containing raspberry polyphenols, the final body weight of rats was similar among all groups (Table 1). The administration of REs significantly increased the pH in the stomach, when compared to the control diet (*p* ≤ 0.05 *vs.* C). However, in the small intestine, except for group E2L, the dietary supplementation with raspberry polyphenols significantly decreased pH of the digesta (*p* ≤ 0.05 *vs.* C). The lowest value of the caecal pH was recorded in the E2L group (*p* ≤ 0.05 *vs.* C). Also in the colon the pH of digesta was significantly lower in all groups treated with raspberry polyphenols as compared to the control animals (*p* ≤ 0.05 *vs.* C). Two-way ANOVA revealed an increase in caecal pH value (*p* = 0.013) in response to a higher level of dietary polyphenols. The ammonia concentrations in the caecal digesta from E1L and E1H groups were significantly lower than in groups with raspberry extract 2 (E2), and at the same time as compared to the C rats (*p* ≤ 0.05 *vs.* C). Moreover, irrespective of the dosage level the addition of E2 significantly increased caecal dry matter concentration *vs.* control group (*p* ≤ 0.05). Except group E2L, the relative colonic digesta mass was lowered by the raspberry polyphenols (*p* ≤ 0.05 *vs.* C). The two-way ANOVA showed that the dietary E2 extract significantly increased the relative mass of colonic digesta when compared to treatment E1 (*p* ≤ 0.05). 

**Table 1:** Body weight (BW) and basic indicators of the gastrointestinal function of rats.

Parameters	Group ^1^					Extract (E) *p*	Dosage (D) *p*	E × D *p*
	C	E1L	E2L	E1H	E2H			
BW, g	271 ± 3.70	275 ± 4.03	274 ± 3.81	269 ± 3.84	273 ± 2.80	0.696	0.305	0.567
Stomach pH	2.38 ± 0.306	3.31 ± 0.211 *	3.68 ± 0.291 *	3.28 ± 0.284 *	3.41 ± 0.220 *	0.324	0.554	0.647
Small intestine:								
Total mass ^2^	2.47 ± 0.037	2.37 ± 0.046	2.36 ± 0.065	2.38 ± 0.057	2.43 ± 0.070	0.801	0.499	0.603
pH	7.19 ± 0.139	6.82 ± 0.083 *	6.98 ± 0.067	6.82 ± 0.076 *	6.76 ± 0.089 *	0.593	0.177	0.176
Caecum								
Tissue ^2^	0.278 ± 0.008	0.269 ± 0.010	0.274 ± 0.012	0.277 ± 0.014	0.276 ± 0.009	0.848	0.677	0.823
Digesta ^2^	1.21 ± 0.081	1.11 ± 0.058	1.07 ± 0.088	1.03 ± 0.104	1.17 ± 0.097	0.584	0.917	0.323
Dry matter (%)	23.0 ± 0.503	24.0 ± 0.612	25.3 ± 0.759 *	24.3 ± 0.634	25.1 ± 0.628 *	0.129	0.969	0.699
Ammonia (mg/g)	0.255 ± 0.014	0.199 ± 0.014 *	0.255 ± 0.007	0.188 ± 0.008 *	0.235 ± 0.016	0.000	0.203	0.742
pH	7.05 ± 0.177	6.62 ± 0.133	6.58 ± 0.092 *	6.72 ± 0.120	7.10 ± 0.121	0.164	0.013	0.080
Colon								
Tissue ^2^	0.437 ± 0.011	0.374 ± 0.018 *	0.398 ± 0.020	0.352 ± 0.013 *	0.378 ± 0.011 *	0.131	0.193	0.974
Digesta ^2^	0.324 ± 0.051	0.270 ± 0.044	0.432 ± 0.048	0.344 ± 0.033	0.407 ± 0.063	0.027	0.619	0.316
pH	7.07 ± 0.129	6.44 ± 0.142 *	6.45 ± 0.098 *	6.29 ± 0.135 *	6.60 ± 0.086 *	0.182	0.994	0.221

Values are expressed as mean ± standard error of mean; E × D, interaction between investigated extracts and dosage of extracts; ^1^ C, was fed a standard diet for laboratory rodents; E1L, E2L were fed diet with low dosage of raspberry extracts characterized by different ratio of the ETs to flavan-3-ols (7.7 and 3.1 respectively); E1H, E2H were fed diet with high dosage of raspberry extracts characterized by different ratio of the ETs to flavan-3-ols (7.7 and 3.1 respectively); ^2^ g/100 g BW; * Data significantly different with the control group at *p* ≤ 0.05 (*t*-Student test).

**Table 2:** Activity of microbial enzymes in the caecum digesta of rats (μmol/h/g).

Parameters	Group ^1^					Extract (E) *p*	Dosage (D) *p*	E × D *p*
	C	E1L	E2L	E1H	E2H			
α-Glucosidase	11.3 ± 1.366	11.0 ± 0.638	11.9 ± 1.058	11.7 ± 0.906	11.6 ± 1.194	0.695	0.826	0.621
β-Glucosidase	1.67 ± 0.118	2.59 ± 0.294 *	2.94 ± 0.383 *	1.57 ± 0.217	1.56 ± 0.298	0.582	0.000	0.556
α-Galactosidase	6.86 ± 0.415	6.77 ± 0.749	6.57 ± 0.556	3.90 ± 0.844 *	4.85 ± 0.660 *	0.602	0.003	0.425
β-Galactosidase	26.9 ± 1.526	26.9 ± 2.722	27.3 ± 6.127	10.9 ± 2.063 *	11.0 ± 1.480 *	0.954	0.000	0.966
β-Glucuronidase	5.48 ± 0.582	5.34 ± 0.599 ^a^	3.85 ± 0.171 ^a,b,^*	2.68 ± 0.284 ^b,^*	4.24 ± 0.878 ^a,b^	0.950	0.051	0.011

Values are expressed as mean ± standard error of mean; E × D, interaction between investigated extracts and dosage of extracts; ^1^ C, was fed a standard diet for laboratory rodents; E1L, E2L were fed diet with low dosage of raspberry extracts characterized by different ratio of the ETs to flavan-3-ols (7.7 and 3.1 respectively); E1H, E2H were fed diet with high dosage of raspberry extracts characterized by different ratio of the ETs to flavan-3-ols (7.7 and 3.1 respectively); ^a,b^ Data with different superscripts in the same column are significantly different with each other at *p* ≤ 0.05 (Duncan test done only in the case of significant E × D interaction); * Data significantly different with the control group at *p* ≤ 0.05 (*t*-Student test).

The activity of microbial enzymes in the caecal digesta is shown in Table 2. In comparison to the control group, the activity of the β-, α-galactosidase in groups with higher amount of the raspberry polyphenols and β-glucuronidase in groups E2L and E1H was significantly decreased. Moreover, the results of a two-way ANOVA analysis of variance indicated dose-depending effects of the raspberry polyphenols on microbial activity. It was observed considerable reduction of the enzymes activity following feeding with higher amount of the raspberry polyphenols, *i.e.*, β-glucosidase and β-, α-galactosidase (*p* ≤ 0.05). The activity of bacterial β-glucuronidase in the caecal digesta was found to be the lowest in group E1H (*p* ≤ 0.05 *vs.* E1L).

The significant extract type by polyphenols dosage interaction for β-glucuronidase activity showed that the aforementioned difference was noted between E1 groups but not between E2 groups. The total caecal concentration of SCFA in rats from E1L group was significantly higher than in the control group, this resulting mainly from the increased concentration of acetic and butyric acids (Table 3, *p* ≤ 0.05 *vs.* C). As compared to the control rats, all groups treated with dietary raspberry polyphenols had higher butyrate concentration in the caecal digesta. The E1L group was associated with significantly higher acetic acid and total SCFA concentrations *vs.* C. In comparison to the control group, the lower concentrations (*p* ≤ 0.05) was noted in the case of propionate (E1H group), isovalerate (both higher dosages), and valerate (E1H group). The extract type by polyphenols dosage interaction was significant for total SCFA (*p* = 0.005), propionate (*p* = 0.049), butyrate (*p* = 0.009) concentrations, and SCFA pool (*p* = 0.006). As for total SCFA concentration and pool, the significant difference between extracts E1 and E2 (E1 > E2) was observed only with regard to the lower but not to the higher dosage of raspberry polyphenols. Moreover, a significant reduction in total SCFA concentration and pool, as well as propionate concentration, followed only the higher dosage of polyphenols from the E1 extract. Such effect was not observed in the case of extract E2. In the case of butyrate, the higher dosage of polyphenols from E2 (but not E1) significantly increased the caecal concentration of that acid.

The profile analysis of three major fatty acids (acetic, propionic, butyric) revealed that diets with dietary raspberry polyphenols were characterized by a significantly higher butric acid/total SCFA ratio (*p* ≤ 0.05 *vs.* C). In groups E1H and E2H the propionic acid/total SCFA and PSCFA/total SCFA ratio were significantly lower in comparison to the control group (*p* ≤ 0.05). The group E2H had a significantly lower acetate share in the SCFA profile as compared to the control animals. Additionally, two-way ANOVA showed that the addition of dietary E2, characterized by a lower ratio of the ellagitannins to flavan-3ols, significantly decreased caecal acetic acid/total SCFA ratio (*p* ≤ 0.05 *vs.* E1 treatment).

As compared to the control group, the plasma lipid profile was affected by both tested raspberry extracts (Table 4). The significant decrease in blood plasma TG concentration followed experimental feeding in all groups with REs, and TC concentration was reduced in groups E1L and E2H (*p* < 0.05 *vs.* C). Except group E2L, all treatments with raspberry polyphenols decreased concentration of HDL cholesterol in the rats’ plasma in comparison to the control group. Two-way ANOVA analysis showed that a higher dosage of raspberry polyphenols significantly lowered value of the HDL profile (*p* < 0.05 *vs.* lower dosage). Moreover, the value of the atherogenic index was significantly decreased in groups E2L and E2H, when compared to the control group (*p* ≤ 0.05).

**Table 3:** Short-chain fatty acids (SCFA) in the caecum digesta of rats.

Parameters	Group ^1^					Extract (E) *p*	Dosage (D) *p*	E × D *p*
	C	E1L	E2L	E1H	E2H			
SCFA, (μmol/g digesta)								
Acetate	42.6 ± 2.080	52.7 ± 2.769 *	40.1 ± 2.210	46.1 ± 2.771	43.8 ± 2.774	0.009	0.592	0.062
Propionate	10.8 ± 0.667	10.7 ± 0.649 ^a^	9.49 ± 1.121 ^a,b^	8.43 ± 0.74 ^b,^*	9.72 ± 0.757 ^a,b^	0.605	0.085	0.049
Isobutyrate	0.451 ± 0.047	0.380 ± 0.073	0.366 ± 0.099	0.772 ± 0.565	0.482 ± 0.138	0.613	0.400	0.647
Butyrate	8.11 ± 1.001	15.7 ± 2.123 ^a,b,^*	11.9 ± 1.714 ^b,^*	11.8 ± 0.887 ^b,^*	16.9 ± 1.385 ^a,^*	0.686	0.720	0.009
Isovalerate	0.435 ± 0.056	0.338 ± 0.074	0.362 ± 0.095	0.190 ± 0.041 *	0.245 ± 0.043 *	0.563	0.058	0.818
Valerate	0.785 ± 0.087	0.864 ± 0.123	0.693 ± 0.089	0.417 ± 0.060 *	0.611 ± 0.080	0.904	0.007	0.055
PSCFA	1.67 ± 0.134	1.58 ± 0.263	1.42 ± 0.253	1.38 ± 0.639	1.34 ± 0.161	0.790	0.707	0.874
SCFA total	63.2 ± 3.833	81.6 ± 3.393 ^a,^*	62.9 ± 3.538 ^b^	67.7 ± 3.995 ^b^	71.8 ± 3.875 ^a,b^	0.058	0.507	0.005
SCFA profile (%)								
Acetate	67.4 ± 1.680	64.6 ± 2.245	64.0 ± 1.930	68.2 ± 1.755	60.8 ± 1.332 *	0.039	0.900	0.080
Propionate	17.2 ± 0.817	14.5 ± 1.079	15.3 ± 1.764	12.5 ± 0.849 *	13.7 ± 1.218 *	0.424	0.173	0.855
Butyrate	12.7 ± 1.131	19.0 ± 2.174 *	18.4 ± 1.782 *	17.5 ± 0.974 *	23.6 ± 1.382 *	0.104	0.274	0.054
PSCFA	2.69 ± 0.264	1.97 ± 0.344	2.35 ± 0.468	1.86 ± 0.744 *	1.89 ± 0.245 *	0.679	0.564	0.727
SCFA pool ^2^	76.8 ± 7.503	90.4 ± 5.821 ^a^	66.5 ± 4.834 ^b^	68.8 ± 7.084 ^b^	84.2 ± 8.391 ^a,b^	0.524	0.769	0.006

Values are expressed as mean ± standard error of mean; E × D, interaction between investigated extracts and dosage of extracts; PSCFA, putrefaction short chain fatty acid; ^1^ C, was fed a standard diet for laboratory rodents; E1L, E2L were fed diet with low dosage of raspberry extracts characterized by different ratio of the ETs to flavan-3-ols (7.7 and 3.1 respectively); E1H, E2H were fed diet with high dosage of raspberry extracts characterized by different ratio of the ETs to flavan-3-ols (7.7 and 3.1 respectively); ^2^ μmol/100g BW; ^a,b^ Data with different superscripts in the same column are significantly different with each other at *p* ≤ 0.05 (Duncan test done only in the case of significant E × D interaction); * Data significantly different with the control group at *p* ≤ 0.05 (*t-*Student test).

**Table 4:** Plasma lipid profile in rats.

Parameters	Group ^1^					Extract (E) *p*	Dosage (D) *p*	E × D*p*
	C	E1L	E2L	E1H	E2H			
TG (mmol/L)	1.270 ± 0.095	0.930 ± 0.080 *	0.924 ± 0.105 *	0.895 ± 0.109 *	0.794 ± 0.111 *	0.605	0.426	0.644
TC (mmol/L)	3.08 ± 0.211	2.34 ± 0.087 *	2.65 ± 0.150	2.67 ± 0.147	2.49 ± 0.177 *	0.666	0.565	0.098
HDL (mmol/L)	1.52 ± 0.092	1.24 ± 0.051 *	1.37 ± 0.070	1.22 ± 0.057 *	1.18 ± 0.102 *	0.565	0.166	0.266
HDL profile ^2^	49.9 ± 1.701	53.3 ± 1.676	52.0 ± 1.943	46.4 ± 2.481	47.5 ± 2.226	0.975	0.012	0.584
AI ^3^	−0.187 ± 0.065	−0.316 ± 0.071	−0.420 ± 0.061 *	−0.352 ± 0.090	−0.429 ± 0.082 *	0.249	0.774	0.869

Values are expressed as mean ± standard error of mean; E × D, interaction between investigated extracts and dosage of extracts; TG, triacylglycerols; TC, total cholesterol; HDL, HDL-cholesterol; AI, atherogenic index; ^1^ C, was fed a standard diet for laboratory rodents; E1L, E2L were fed diet with low dosage of raspberry extracts characterized by different ratio of the ETs to flavan-3-ols (7.7 and 3.1 respectively); E1H, E2H were fed diet with high dosage of raspberry extracts characterized by different ratio of the ETs to flavan-3-ols (7.7 and 3.1 respectively); ^2^ % of TC; ^3^ log(TG/HDL); * Data significantly different with the control group at *p* ≤ 0.05 (*t*-Student test).

## 3. Discussion

In this study, the E1 and E2 raspberry extracts obtained from pomace were consisted of 33.8 and 63.3 g/100 g phenolic compounds, respectively, most of which were ETs (29.7 and 47.7 g/100 g, respectively) and flavan-3-ols (3.8 and 15.2 g/100 g, respectively). The range of the total content of phenolic compounds in fresh raspberry in other studies was around 0.234 g/100 g [18] and 0.714 g/100 g [19]. Furthermore, the key polyphenols present in the fresh raspberry *i.e.*, ETs are between 0.119–0.323 g/100 g and for total anthocyanins have been reported from 0.002 to 0.325 g/100 g [19]. It is worth to mention, that during processing of the raspberries most of the anthocyanins stayed in the juice, therefore polyphenol extracts from pomace contained lower concentration of these bioactive compounds. The concentrations of bioactive substances in raspberry are strongly influenced by many factors, such as variations in genotype, climate, harvest season, temperature, and the degree of ripeness [18,20]. To represent a realistic amount of fresh raspberries consumed by humans, the diets used in this study were calculated with the aid of the body surface area normalization method [21] and literature data for polyphenol content in the raspberry. The polyphenols dietary treatment was determined to correlate to a daily consumption of fresh raspberry by an adult weighing 70 kg in amount of 0.095−0.291 kg and 0.19−0.582 kg when compared with diet supplemented with E1 and E2 raspberry extract, respectively.

After eight weeks feeding period with raspberry polyphenols, the rats’ growth was similar to that of the control group. However, changes in the basic gastrointestinal tract indicators were observed in all examined groups. The difference lies in a significant decrease of the pH value in the digesta of the small intestine and colon. The acidification of the digesta promotes positive microbiota proliferation and decreases the growth of pathogenic bacteria species [22]. In the caecum the acidification of the digesta was observed only in groups with low dosage of the raspberry polyphenols. Opposite effect was observed in stomach when pH value was significantly higher after administration of the examined raspberry polyphenols. The main polyphenolic compounds of both raspberry extracts were ETs, and it has been reported that this compound releases ellagic acid (EA) upon hydrolysis *in vivo* [23]. Therefore, the elevation of the pH can be explained by the poor solubility of EA in water and its ionization at physiological pH to form a complex with alkaline calcium and magnesium ions in the intestine [24]. Furthermore, the poor solubility of EA may result in lower amount of bounded water and higher amount of the dry matter in the caecal digesta. In the present nutritional experiment, only diets with E2 significantly increased amount of the dry matter in the caecal digesta.

Some studies have shown that polyphenols in the diet, especially ETs and proanthocyanidins, may have modulated the activity of the microbiota and digestive enzymes [7,11,25,26]. Larrosa *et al.* [27] found that ETs from pomegranate decreased enterobacteria and increased lactobacilli as well as bifidobacteria. It can be assumed that ETs and proanthocyanidins in the diet could selectively modulate the composition of the microbiota and thus decrease potentially harmful species of bacteria which are responsible e.g., the enhancing activity of microbial β-glucuronidase which may involves in the generation of toxic and carcinogenic metabolites in the hindgut [7,16]. In present study, activity of microbial β-glucuronidase was the lowest in group E1H which contained higher ratio of the ETs to flavan-3-ols. The same effect of the ETs was observed in previous study on rats fed with diet contained strawberry extract [11]. McDougall *et al.* [28] reported that polyphenol-rich extracts from berries inhibit α-amylase and sucrose *in vitro*. It is also suggested that ETs and proanthocyanidins were potent inhibitors of amylase [29]. In this study, administration of diets with examined raspberry polyphenols had no effect on the activity of the α-glucosidase. Furthermore, raspberry extracts used in this study were also a source of flavonoids which applied as a dietary supplement may decrease the activity of bacterial β-glucosidase as well as β- and α-galactosidases in the caecal digesta of rats [30]. Indeed, higher dosage of the examined raspberry polyphenols considerable reduced activity of microbial β- and α-galactosidase in the caecal digesta when compared with lower dosage of these extracts. These microbial enzymes are assisting during hydrolysis of indigestible oligosaccharides, therefore lower activity of galactosidases may increase colonic fermentation and gas production which is undesired especially for people with irritable bowel syndrome [31]. On the other hand, some authors provided information that β-galactosidase could be responsible for creating detrimental metabolites, e.g., from kaempferol-3-*O*-galactoside-rhamnoside-7-*O*-rhamnoside [32]. The inhibitory effects of plant phenols can be caused by their covalent attachment to reactive nucleophilic sites in an enzyme such as free amino and thiol groups or tryptophan residues [33]. The consumption of the diets with raspberry polyphenols contained higher ratio of the ETs to flavan-3-ols beneficially increased ammonia utilization. The decreased ammonia concentration is considered a positive change because this compound can destroy cells, alter nucleic acid synthesis, induce cancerous cell growth and increase virus infections in the lower bowel [34].

The modulation of the microbial activity was also spotted by changes in the concentration and, in some cases, the pool of SCFAs which could be reliable markers of the extent of fermentation occurring in large intestines by colonizing microbiota [35]. The SCFAs have positive health effects, especially butyric acid which has been shown to be anti-inflammatory, to modulate oxidative stress and to be a main energy substrate for colonocytes. Similar effects has been linked to propionic acid, but generally to a lesser extent, while acetic acid is associated with fewer physiological effects [36,37]. An increased content of polyphenols in the diet may reduce the abundance of gastrointestinal microflora and thereby decreased the production of SCFA [11,30]. Indeed, from all experimental groups E1L contained lower concentration of the raspberry polyphenols showed the highest concentration of the total SCFA mostly acetate and butyrate. All groups with raspberry polyphenols contributed to beneficial increased production of the butyric acid in the caecum in comparison to the control group. The same result was spotted in the pool of SCFA. Furthermore, the calculated SCFA profile showed that higher dose of raspberry polyphenols significantly reduced value of the putrefactive short-chain fatty acids (PSCFA) which may suggest less intensive anaerobic bacterial fermentation of polypeptides and amino acids [38]. Significant reduction of the PSCFA concentration in the caecum digesta was also observed in study on Wistar rats fed diets contained high level of the ETs extracted from strawberries [7]. 

Above changes in the caecal concentrations of SCFA might be associated to some extent with blood markers of the lipids profile in the rats. Acetate and propionate are known to differentially influence fat metabolism; acetate is the primary substrate for cholesterol synthesis in the liver, whereas propionate has been reported to be involved in the cholesterol lowering effect [14,39]. In this study, higher dosage of the raspberry polyphenols to the diets significantly reduced value of the HDL profile in the plasma when compared with groups contained lower dosage of these dietary compounds. Furthermore, studies with animal models demonstrate that supplementation with foods containing polyphenols, basically with proanthocyanidins may favorable reduced concentration of the total cholesterol, LDL-cholesterol, TG and increased HDL-cholesterol in the plasma [7]. In present study, raspberry polyphenols in group E1L and E2H decreased value of the total cholesterol in comparison to the control group. Moreover, when compared with control group, raspberry polyphenols in all groups beneficially reduced concentration of the TG. Nevertheless, only in group E2L with lower ratio of the ETs to flavan-3-ols was not considerable reduced concentration of the HDL-cholesterol in rat’s plasma and therefore also the value of the atherogenic index was significantly lower in groups E2L and E2H. This may suggest a higher biological activity of the raspberry flavan-3-ols with high concentration of the proanthocyanidins than ETs.

## 4. Materials and Methods 

### 4.1. Raspberry Phenolic Extracts

The fresh raspberry press cake was taken from the concentrated juice production line of the Alpex Company (Łęczeszyce, Poland). Extraction of phenolics was carried out according to the following procedure: fresh press cake (5 kg) was extracted with 60% acetone with a 1:5 (*w*/*v*) ratio of solvent to plant material. This process was facilitated with shaking-assisted extraction 150 rpm (Orbital Shaker DOS-10L, Elmi, Aizkraukles, Riga, Latvia) and was conducted at room temperature for 8 h. The extract was collected by soaking on filtration cloth, and the residue were extracted again with 60% acetone in the same condition as above. The extracts obtained in the two successive steps were combined and filtered on Hobrafit S40 N-5 µm nominal retention, 3.6-mm thickness cellulose sheet (Hobra-Školnik S.R.O., Broumov, Czech Republic). From the resulting extract acetone was removed using vacuum rotary evaporator Basis Hei-VAP HL (60 °C, 450–72 mbar; Heidolph, Schwabach, Germany), and the extract was divided in two equal volumes (~10L). The first was concentrated, using vacuum rotary evaporator Basis Hei-VAP HL (Heidolph, Schwabach, Germany) to approx. 15 Brix, and next was freeze-dried (−32 °C, 48 h; Christ, Alpha 1-2 LDplus, Osterode am Harz, Germany)—Extract 1. The second was purified on 100 × 11 cm glass column filled with Amberlite XAD 1600 (DOW, Midland, MI, USA). The extract was applied at a flow rate of 150 mL/min. Afterwards, the column was washed with 10L of 10% ethanol, and eluted with 10L of 40% ethanol and 10 L of 70% ethanol. Fraction eluted with 40% ethanol, characterized with high concentration of ellagitannins, was next subjected to removal of ethanol using vacuum rotary evaporator Basis Hei-VAP HL (60°C, 450–72 mbar; Heidolph) and freeze-drying (−32 °C, 48 h; Christ, Alpha 1-2 LDplus)—Extract 2.

### 4.2. Basic Chemical Composition

Dry matter, ash, crude protein, crude fat and, total dietary fibre (TDF) were determined according AOAC official methods [40]: 920.151; 940.26; 920.152; 930.09; 985.29, respectively. Carbohydrates were determined using formula: Carbohydrate = total solids – (protein + fat + ash). 

For ellagitannins and anthocyanins measurements, the samples of raspberry phenolics extracts, and known standards were diluted with 50% (*v*/*v*) methanol, filtered through PTFE filters (0.45 µm) and introduced into high performance liquid chromatography (HPLC) systems.

#### 4.2.1. Quantification of Ellagitannins

The content of ellagitannins were determined using a Smartline chromatograph (Knauer, Berlin, Germany) composed of a degasser (Manager 5000), two pumps (P1000), autosampler (3950), thermostat and detector PDA (2800). The ellagitannins were separated on a 250 x 4.6 i.d., 5 µm, Gemini C18 110A column (Phenomenex, Torrance, CA, USA) by gradient elution with 0.05% (*v*/*v*) phosphoric acid in water (solvent A) and 83:17 (*v*/*v*) acetonitrile:water with 0.05% phospohoric acid (solvent B). The column temperature was set to 35 °C, the flow rate was 1.25 mL/min, and the gradient program was as follow: 0–5 min, 5% (*v*/*v*) B; 5–10 min, 5%–15% (*v*/*v*) B; 10–35 min, 15%–40% (*v*/*v*) B; 35–40 min, 40%–73% (*v*/*v*) B; 40–44 min, 73% (*v*/*v*) B; 44–46 min, 73%–5% (*v*/*v*) B; 46–54 min, 5% (*v*/*v*) B. The injection volume was 20 µL. Data were collected using the ClarityChrom version 3.0.5.505 Program (Knauer). Ellagitannins were detected at 250 nm, and the standard curves using lambertianin C, sanguiin H-6, and ellagic acid were used for quantification. For unidentified ellagotanins, sanguiin H-6 curve were used for quantification. Standards of lambertianin C and sanguine H-6 were isolated from raspberry extract as described by Sójka *et al.* [41]. Ellagic acid standard was purchased from Extrasynthese (Genay, France).

#### 4.2.2 Quantification of Flavan-3-ols

The content of flavan-3-ols *i.e.*, sum of proanthocyanidins and catechins were determined using the method described by Sójka *et al.* [41]. For separation the same column and conditions were used. For this analysis a Shimadzu (Tokyo, Japan) system equipped with a pump (LC-20AD), degasser (DGU-20A5R), autosampler (SIL-20ACHT), thermostat (CTO-10ASUP), detector (RF-10AXL), and LabSolutions Lite version 5.52 software were used.

#### 4.2.3. Quantifications of Anthocyanins

HPLC coupled with a DAD and an electrospray ion (ESI) trap mass spectrometer was used for the identification of anthocyanins. The HPLC system was equipped with a SCM1000 membrane solvent degasser (ThermoQuest, San Jose, CA, USA), a binary high pressure gradient pump (1100 Series; Agilent Technologies, Santa Clara, CA, USA), autosampler, and a column oven (Surveyor Series, Thermo-Finnigan, San Jose, CA, USA). A Gemini C18 110A 250 mm × 4.6 mm i.d. (Phenomenex, Torrance, CA, USA) 5 µm column was used. The column temperature was 30 °C and the injection volume was 10 µL. The chromatographical data were collected using Xcalibur software, version 1.2 (Thermo-Finnigan). The solvents and the gradient used for anthocyanin separations were as follows: solvent A, 0.25% (*v*/*v*) formic acid in water; solvent B, 0.25% (*v*/*v*) formic acid in acetonitrile, the flow rate was 12 mL/min; the gradient programme (time min–% (*v*/*v*) B): 0–5, 2–5, 32–20, 37–70, 42–70, 45–5, and 55–5.

The MS system coupled to the HPLC was an LCQ DECA ion trap mass spectrometer (Thermo-Finnigan) equipped with an ESI source used in the negative mode. The source parameters were as follows: ion spray voltage, 4.50 kV; capillary voltage, −23 V; capillary temperature, 240 °C; and sheath nitrogen gas flow rate, 80 (arbitrary units).

The phenolics were quantified using a KNAUER Smartline chromatograph equipped with a degasser (Manager 5000), two pumps 1000, autosampler 3950, column oven Jetstream Plus 2 and detector PDA 2800. The separation and quantification were determined in the same conditions described by Sójka *et al.* [42]. The chemical and polyphenolic compositions of the raspberry extracts are presented in Table 5 and Table A1.

**Table 5:** Phenolic fractions of raspberry extracts.

Compound (mg/100 g)	Extract 1 (E1)	Extract 2 (E2)
Ellagitannins		
Ellagitannin derivatives *	2070.2 ± 103.1	4774.7 ± 110.2
Lambertianin C	15406.9 ± 407	22500.1 ± 414.3
Sanguiin H-6	11464.3 ± 11.4	20427.4 ± 306.0
Ellagic acid	804.1 ± 23.6	215.8 ± 23.6
Total ellagitannins	29745.5 ± 399.9	47702.2 ± 881.8
Flavan-3-ols		
(+)-Catechin	79.1 ± 1.6	186.0 ± 2.8
(−)-Epicatechin	238.7 ± 8.1	4352.4 ± 62.6
Procyanidins	3528.9 ± 84.2	10713.2 ± 1037.7
Terminal units (%)		
(+)-Catechin	2.4 ± 0.1	3.9 ± 0.2
(−)-Epicatechin	49.6 ± 0.4	54.0 ± 0.8
Extension units (%)		
(+)-catechin	45.8 ± 0.3	40.0 ± 1.3
(−)-Epicatechin	2.3 ± 0.0	2.1 ± 0.0
mDP	1.93 ± 0.0	1.73 ± 0.0
Total flavan-3-ols	3846.8±77.7	15251.6 ± 1103.1
Anthocyanins		
Cyanidin-3-sophoroside **	100.9 ± 0.9	241.3 ± 0.3
Cyanidin-3-glucosyl- rutinoside **	12.5 ± 0.1	49.0 ± 0.6
Cyanidin-3-glucoside	76.2 ± 0.9	63.2 ± 0.4
Cyanidin-3-rutinoside **	9.8 ± 0.0	21.0 ± 0.2
Pelargonidin-3-glucoside **	4.3 ± 0.0	2.7 ± 0.2
Total anthocyanins	203.6 ± 1.7	377.2 ± 0.8
Total polyphenols	33795.1 ± 479.3	63331 ± 1985.7

Values are expressed as mean ± standard deviation (*n* = 3); mDP, mean degree of procyanidin polymerization; * The content calculated based on sanguiin H-6 standard; ** The content calculated on cyaniding-3-glucoside standard.

### 4.3. Animal Study

**Figure 1:**
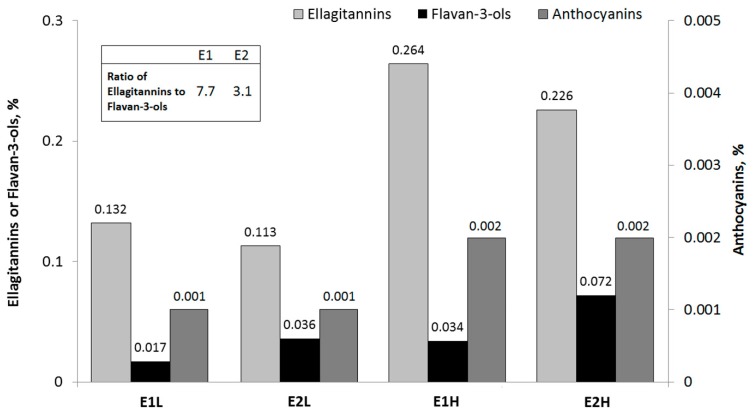
Concentration of polyphenols in diets with raspberry extracts. E1L, E2L were fed diet with low dosage of raspberry extracts characterized by different ratio of the ETs to flavan-3-ols (7.7 and 3.1 respectively); E1H, E2H were fed diet with high dosage of raspberry extracts characterized by different ratio of the ETs to flavan-3-ols (7.7 and 3.1 respectively); E1, diet with raspberry extract 1; E2, diet with raspberry extract 2.

This study was carried out in strict accordance with the recommendations of the National Ethic Commission (Warsaw, Poland). All procedures and experiments complied with the guidelines and were approved by the Local Ethic Commission of the University of Warmia and Mazury (Olsztyn, Poland, Permit Number: 32/2012) with respect to animal experimentation and care of animals under study, and all efforts were made to minimize suffering. To determine the number of animals in group, the sample size estimation was done according to the approaches proposed by Dell *et al.* [43]. The nutritional experiment was performed on 40 male Wistar rats allocated to five groups of eight animals each, and the rats were housed individually in plastic cages. The initial body weight was comparable among groups and equaled 120.1 ± 1.72 g. For 8 weeks, each group was fed a modified version of the semi-purified rodent diet recommended by Reeves [44]. All experimental diets were similar in terms of dietary ingredients except for the phenolic fraction. The rats were fed with a standard diet (group C) or its modification with two types of raspberry extracts (group E1 and E2) characterized by different ratio of ellagitannins to flavan-3-ols (7.7 for E1 and 3.1 for E2) and added to a diet at two dosages of polyphenolic compounds (0.15 and 0.30% of a diet; L and H treatments, respectively). In all experimental diets, the raspberry phenolic extracts were added at the expense of corn starch. Details about the proportional composition of each group-specific diet are shown in Table A2 and Figure 1. The duration of the experiment and dose of the extracts in diets were adjusted according to the experience from previous studies with polyphenols [11,13,17]. The individual body weights of rats and food intakes were recorded on a weekly and daily basis, respectively. The animals were maintained under standard conditions at a temperature of 21–22 °C and a relative air humidity of 50%–70% with intensive room ventilation (15×/h) and a 12 h lighting regimen.

### 4.4. Sample Collection and Analysis

At the termination of the experiment, the rats were anesthetized with sodium pentobarbital according to the recommendations for euthanasia of laboratory animals (50 mg/kg body weight). After laparotomy, blood samples were collected from the *vena cava* and stored in tubes containing ethylenediaminetetraacetic acid. Immediately after euthanasia (*ca.* 10 min) the stomach, small intestine, caecal and colonic pH values were measured directly in the intestine segments (model 301; Hanna Instruments, Vila do Conde, Portugal). The small intestine, caecum and colon were removed and weighed. Fresh caecal digesta was used for the determination of ammonia content, which was extracted, trapped in a solution of boric acid, and quantified by direct titration with sulfuric acid [45]. The concentrations of SCFAs in ceacal digesta samples were determined by gas chromatography (GC-2010, Shimadzu, Kyoto, Japan). The samples (0.2 g) were mixed with 0.2 mL of formic acid, diluted with deionized water and centrifuged at 7211 *g* for 10 min. The supernatant was loaded onto a capillary column (SGE BP21, 30 m × 0.53 mm) using an on-column injector. The initial oven temperature was 85 °C, it was raised to 180 °C at 8 °C min^−1^ and held for 3 min. The temperature of the flame ionization detector and the injector was 180 °C and 85 °C, respectively. The sample volume for GC analysis was 1 μL. The concentrations of caecal putrefactive SCFAs (PSCFAs) were calculated as the sum of iso-butyric acid, isovaleric acid and valeric acid. The caecal SCFA pool size was calculated as the sum of SCFA concentration in the digesta and caecal digesta mass. All SCFAs analyses were performed in duplicate. Pure acetic, propionic, butyric, isobutyric, isovaleric and valeric acids were obtained from Sigma Co. (Poznan, Poland), and their mix was used to create a standard plot and then to calculate the amount of single acids. This additional set of pure acids was included in each GC run of samples at five sample intervals to maintain calibration. The activity of selected bacterial enzymes (α- and β-glucosidase as well as α-, β-galactosidase and β-glucuronidase) released into the caecal environment was measured by the rate of *p*- or *o*-nitrophenol release from their nitrophenyl glucosides according to a previously described method [38].

The blood was centrifuged for 15 min at 380 g and 4 °C, and the obtained plasma was then stored at −70 °C until analysis. The plasma concentration of triglycerides (TG), total cholesterol (TC) and HDL cholesterol fraction (HDL-C), were estimated using reagents from Alpha Diagnostics Ltd. (Warsaw, Poland). Based on the plasma lipid profile, the atherogenic index (AI) was calculated as previously described using the following formula: lg(TG (mmol/L)/HDL-C (mmol/L)) [46]. 

### 4.5. Statistical Analysis

STATISTICA software (version 10.0; StatSoft Corp., Krakow, Poland) was used to determine whether variables differed among the treatment groups. A two-way repeated measures ANOVA was applied to assess the effects of the two types of the raspberry extracts (diet with raspberry extract 1 or 2; E), the dietary dosage of the raspberry polyphenols (D) and the interaction between these investigated factors (E × D). If the ANOVA revealed a significant interaction (*p* ≤ 0.05), the differences between the individual groups were then assessed with Duncan’s multiple range post hoc test at *p* ≤ 0.05. Differences between groups with raspberry polyphenols and control group were analyzed statistically using unpaired two-tailed Student’s t-test at a significance level of *p* ≤ 0.05. The results are presented as the mean values ± standard error of the mean (SEM), except for the phenolic composition of raspberry extracts which are expressed as the mean and the standard deviation (SD) of the mean.

## 5. Conclusions 

Irrespective of polyphenol dietary levels, both examined raspberry extracts obtained from pomace exerted an additional favorable effect on microbial functioning by reducing activity of the β-glucuronidase and elevating production of the butyric acid in the caecum as well as on lipid metabolism by reducing TG in rat’s plasma. When compared both raspberry extracts, E1 with higher ratio of the ETs to flavan-3-ols more effectively reduced concentration of the ammonia and elevated production of acetate in the caecum digesta than E2. On the other hand, only E2L did not considerably reduce the concentration of HDL-cholesterol in rat plasma and therefore the value of the corresponding atherogenic index was significantly lower in groups with E2 when compared with control group. Furthermore, the results of a two-way analysis of variance indicated dose-dependent effects of the raspberry polyphenols on microbial activity and blood parameters. In diets with higher amount of the raspberry polyphenols a considerable reduction of the β- and α-galactosidase activity in caecum digesta as well as lower values of the HDL profile than in the groups with the lower dosage of these polyphenols were observed. To conclude, the examined raspberry extracts are a valuable source of ETs and flavan-3-ols which may favorably modulate the activity of the caecal microbiota and blood lipid profile in rats; however, the intensity of these effects may be related to the dosages of dietary polyphenols as well as to their profile, e.g., the ETs to flavan-3-ols ratio. Furthermore, this experiment was performed on an animal model, therefore the use of the polyphenolic raspberry extracts as a functional additives to food should be also verified by human study.

## Appendix A

**Table A1:** Basic compounds of the raspberry extracts.

Compound (%)	Extract 1 (E1)	Extract 2 (E2)
Dry matter	98.8	98.4
Crude ash	5.2	0.3
Crude protein	6.8	2.2
Crude fat	0.2	0.1
Carbohydrates	49.8	27
Total dietary fiber	2.4	8
Total polyphenols	33.8	63.3

**Table A2:** Composition of the group-specific diets.

Ingredient (%)	Group ^1^				
	C	E1L	E2L	E1H	E2H
Casein	14.8	14.8	14.8	14.8	14.8
d,l-methionine	0.2	0.2	0.2	0.2	0.2
α-Cellulose	5	5	5	5	5
FOS	3	3	3	3	3
Soy oil	8	8	8	8	8
Cholesterol	0.5	0.5	0.5	0.5	0.5
Mineral mix ^2^	3.5	3.5	3.5	3.5	3.5
Vitamin mix ^2^	1	1	1	1	1
Extract1	-	0.45	-	0.90	-
Extract2	-	-	0.24	-	0.48
Corn starch	64	63.55	63.76	63,1	63.52
Polyphenols (%)	-	0.15	0.15	0.30	0.30

^1^ C, was fed a standard diet for laboratory rodents; E1L, E2L were fed diet with low dosage of raspberry extract contained 33.8 g/100 g and 63.3 g/100 g polyphenols, respectively; E1H, E2H were fed diet with high dosage of raspberry extract contained 33.8 g/100 g and 63.3 g/100 g, polyphenols, respectively; ^2^ Recommended for the AIN-93G diet [44].
